# Impact of Match Violations on Applicants’ Perceptions and Rankings of Residency Programs

**DOI:** 10.7759/cureus.12823

**Published:** 2021-01-20

**Authors:** Reesa L Monir, Kristina Michaudet, Joseph G Monir, Kiarash P Rahmanian, Charlie Michaudet, Lou Ann Cooper, Heather Harrell

**Affiliations:** 1 Dermatology, University of Florida College of Medicine, Gainesville, USA; 2 Radiology, Stanford University School of Medicine, Stanford, USA; 3 Orthopedics and Rehabilitation, University of Florida College of Medicine, Gainesville, USA; 4 Emergency Medicine, Central Carolina Hospital, Sanford, USA; 5 Family Medicine, Stanford Healthcare, Menlo Park, USA; 6 Medical Education, University of Florida College of Medicine, Gainesville, USA; 7 Internal Medicine, University of Florida College of Medicine, Gainesville, USA

**Keywords:** medical school, residency, nrmp, match, violations, applicants, interviews

## Abstract

Introduction

The National Resident Matching Program (NRMP) requires all Match participants to adhere to a strict code of conduct known as the Match Participation Agreement, yet Match violations continue to occur. We sought to determine how interview experiences, including Match violations, impact applicants’ perceptions and rankings of residency programs.

Methods

An electronic survey was sent to all accredited medical school Deans of Student Affairs and Association of American Medical Colleges Student Representatives for distribution to fourth-year medical students. Questions assessed pressures that residency programs placed on applicants during interview season and their impact on applicants. Both quantitative and qualitative data were collected.

Results

Of the 433 included respondents, 31.2% (n = 135) reported breaches of the NRMP Match Participation Agreement. Of those, 63% (n = 85) had a negative perception of the violating programs, and 37.8% (n = 51) were less likely to rank those programs highly. Violations included asking applicants about the locations of their other interviews (60.3%, n = 261), pressuring applicants to reveal their ranking (24.0%, n = 104), explicitly requesting applicants to reveal their ranking (6.5%, n = 28), asking applicants to provide a commitment before Match day (3.9%, n = 17), and other behavior that was felt to ignore the spirit of the Match (16.4%, n = 71). Implying that applicants would match into a program if they ranked it highly (37.2%, n = 161) was received positively by 65.2% (n = 105) of applicants experiencing this breach, with 42.2% (n = 68) ranking the program more highly. Three major themes impacting applicants’ impressions of residency programs emerged from the qualitative data: interview experience, professionalism, and post-interview communication (PIC). Respondents overwhelmingly agreed that PIC should either be eliminated or that programs should set clear expectations for PIC.

Conclusions

Match violations continue to occur, despite the NRMP Match Participation Agreement. With the notable exception of communication implying that applicants would match into a program, applicants overwhelmingly view programs that commit these violations negatively and often rank these programs lower as a result.

## Introduction

One of the most important and daunting tasks facing medical students is obtaining a residency position at their preferred program. Likewise, residency programs have a stake in securing highly desirable candidates. It is therefore unsurprising that the application process has historically been fraught with attempts by both programs and applicants to influence each other’s decisions. These tensions culminated in the late 1940s, with hospitals offering residency positions to students prior to their fourth year and giving them as little as 12 hours to make vital career decisions [1]. In an attempt to neutralize these so-called “exploding offers” and to bring order to an increasingly chaotic process, the National Residency Match Program (NRMP) was created in 1952 [2].

In an effort to maintain fairness and avoid the pressure that programs historically placed on applicants, the NRMP requires both residency programs and applicants to adhere to a strict code of conduct [3]. Programs found to be in violation are subject to penalties as severe as complete expulsion from the Match [4]. Despite this, recent literature indicates that Match violations continue to occur with significant frequency [5-7]. However, there is a paucity of data regarding the effect of violations on applicants. The goal of this study is to fill this gap in the literature by determining how interview experiences, particularly Match violations, affect applicants’ perceptions and final rankings of programs.

## Materials and methods

Data collection 

We collected data using an electronic survey that contained demographic data as well as forced response and open-ended questions. We referenced prior surveys from similar studies [5,7] and sought input from medical students, residents, and faculty at the University of Florida College of Medicine (UFCOM) when designing the survey. Students who provided survey feedback did not take part in the study. 

The Dean of Student Affairs and the Association of American Medical Colleges (AAMC) Student Representative for the UFCOM distributed the electronic survey to their counterparts at all US Liaison Committee on Medical Education (LCME)-accredited medical schools, who were asked to disseminate the survey to their fourth-year medical students. A reminder email was sent four weeks later. We collected responses for 11 weeks following the day after rank list submission via an anonymous Research Electronic Data Capture (REDCap) link (Vanderbilt University, Nashville, Tennessee, United States). Respondents who did not complete all Likert questions were excluded from analysis. The University of Florida Institutional Review Board (IRB) approved this study as exempt.

Data analysis 

We computed descriptive statistics and conducted bivariate analyses with several cohorts of respondents including those who indicated that they experienced Match violations versus those who did not. Additionally, we stratified by demographic variables such as Step 1 score and number of interviews. The bivariate analyses included were Fisher’s exact tests and Pearson product-moment correlation coefficients. We computed correlations between interview pressures and demographic variables. We conducted multivariate regression analysis in forced inclusion models. Significance was defined as p < 0.05. SAS V.9.4 for Windows (Cary, North Carolina, USA) was used for analysis.

Two of our investigators (KM and RLM) independently grouped the free-response comments into general themes and then collaboratively merged the themes. Two different investigators (HH and LAC) further refined these themes in order to characterize all comments in the most inclusive manner possible. For the purpose of grouped analysis, specialties participating in the NRMP Match were divided into “more competitive” and “less competitive” groups based on historic match rates. The “more competitive” group consisted of dermatology, neurological surgery, orthopedic surgery, plastic surgery, and vascular surgery, and the “less competitive” group consisted of all other specialties.

## Results

A total of 503 students responded to the survey. Eighty-six percent (n = 433) of respondents completed all Likert questions and were included in the analysis. Approximately one-third of respondents completed the free-response questions (27.0%-31.2%, n = 117-135), with slight variability as some students left certain free-response questions blank.

Respondent characteristics 

Fifty-three percent (n = 231) of included respondents were females, 46% males (n = 199), and 0.6% (n = 3) others. Men and women had statistically significant differences in only two survey items. Men were more likely to have Step 1 scores greater than 240 (45.7% of men vs. 33.8% of women, p = 0.04). Women were more likely than men to have a negative or very negative impression of a program after being asked about other interview locations (59.7% of women vs. 45.8% of men, p = 0.03). 

Most survey participants attended public medical schools (59.1%, n = 256), were located in the Southern region (54.7%, n = 237), and were applicants to non-surgical specialties (74.1%, n = 321). Many participants were top scorers with > 240 on Step 1 (39.5%, n = 171) and > 250 on Step 2 (47.8%, n = 207). Seventy-seven percent (n = 332) completed at least 11 interviews for a categorical or advanced position. 

Applicants from the various regions were comparable in terms of gender (p = 0.56), medical school type (p = 0.07), Step 1 score (p = 0.24), and Step 2 score (p = 0.66). There was a statistically significant difference in the number of respondents applying to medical vs. surgical specialties among the different regions (p = 0.003), with the fewest respondents from surgical specialties in the Western region (13.6%, n = 9) and the most from the Central region (29.9%, n = 26). The Western region had fewer Alpha Omega Alpha Medical Honor Society (AOA) members (p < 0.0001), fewer Gold Humanism Honor Society (GHHS) members (p < 0.0001), and significantly more students reporting their class rank as unknown (p < 0.0001). Although the majority of applicants in all regions reported 11 or more interviews, there was a statistically significant difference in the number of applicants having greater than or equal to 11 interviews among the regions, with 95.3% of respondents in the Northeast having this number compared with an average of 76.3% for all other regions (p = 0.0009).

Quantitative results

Overall, 31.2% (n = 135) of applicants reported experiencing breaches of the NRMP 2018 Match Participation Agreement, and 28.9% (n=39) experienced four or more breeches. Applicants completing at least 11 interviews were more likely to experience Match violations than those completing fewer than 11 (85.9% vs 14.1%, p = 0.003). Step 2 score of > 250 was associated with higher rates of reported match violations (p = 0.03). However, Step 2 score is confounded by this group also being more likely to have greater than 11 interviews (p < 0.0001), a factor which was independently associated with higher reports of match violations (p = 0.003). Multivariate analysis showed that, when controlling for other applicant factors in the model, Step 2 score was not independently predictive of Match violations (see Multivariate Analysis below). Applicants to more competitive specialties were more likely to report match violations than their peers in less competitive specialties, a finding that approached but did not reach statistical significance (50% vs. 30%, respectively, p = 0.06). No other applicant characteristics were predictive of reporting a violation.

In response to being asked who committed match violations, 95.6% of respondents identified program directors, 84.4% other faculty, 47.4% residents, and 6.7% administrative staff. The most common violation was asking applicants about the locations of their other interviews (60.3%, n = 261). The majority of applicants who experienced this behavior were left with a negative to very negative impression of the programs (53.3%, n = 139), and 22.6% (n = 59) were less to much less likely to rank the programs highly. The next most common violation was pressure to provide statements of interest to programs outside of the applicant’s geographic region (46.7%, n = 202), which had no impact on applicants’ impression of a program in 74.8% of cases (n = 151) and a negative to very negative impression of the program in a sizable minority of cases (22.3%, n = 45). This negatively impacted a program’s final ranking in 10.9% of cases (n = 22) and had no effect in 84.2% of cases (n = 170).

Other violations included implying that applicants would match into a program if they ranked it highly (37.2%, n = 161), pressuring applicants to reveal their ranking (24.0%, n = 104), explicitly requesting applicants to reveal their ranking (6.5%, n = 28), and asking applicants to provide a commitment before Match Day (3.9%, n = 17). The majority of applicants who either received an explicit request to reveal ranking to a program before rank list submission or who were asked to provide a commitment to a specific program before Match Day had a negative to very negative impression of that program in 71.4% (n = 20) and 41.2% (n = 7) of cases, respectively. These applicants were also less to much less likely to rank that program highly in 50% (n = 14) and 47.1% (n = 8) of cases, respectively. Conversely, 65.2% (n = 105) of applicants had a positive to very positive impression of a program, and 42.2% (n = 68) ranked the program more to much more highly if they were told they would match at that program if said program was ranked highly on their list. 

Finally, 16.4% (n = 71) of applicants experienced other behavior on the part of residency programs that they felt ignored the spirit of the Match. This behavior was defined as unethical behavior, pressure to attend a second-look visit, inappropriate post-interview communication (PIC), or other undue pressure. Of those 71 applicants, 74.6% (n = 53) had a negative to very negative impression of the program, and 57.7% (n = 41) were less to much less likely to rank it highly. The majority of these applicants (49.3%, n = 35) felt the spirit of the Match was ignored two to three times during interview season, while 11.3% (n = 8) of applicants reported it was ignored at least six times. Table 1 summarizes the residency program actions and applicant reactions in terms of their impact on impression of programs and program ranking.

**Table 1:** Impact of Interview Experiences on 2018 Applicants’ Impressions and Ranking of Programs (n = 433) Table 1 summarizes eight actions on the part of residency programs as well as the total number and percentage of respondents who experienced these actions (out of 433 study participants). For each program action, we asked the participants responding “yes” to rate on a Likert scale how that action impacted their impression of the program (very negatively/negatively, no impact, very positively/positively) and how it affected their actual ranking of that program (much less/less likely to rank it highly, no effect, or much more/more likely to rank it highly).

Residency Program Actions	Total No. (%) of Events	Applicant Reactions	Likert	No. (%) of Applicants
Pressure to reveal ranking	104 (24.0%)	Impact on impression of program	Very negatively/negatively	46 (44.2%)
			No impact	55 (52.9%)
			Very positively/positively	3 (2.9%)
		Effect on actual ranking	Much less/less likely to rank it highly	33 (31.7%)
			No effect	56 (53.8%)
			Much more/more likely to rank it highly	15 (14.4%)
Explicit request to reveal ranking before rank list submission	28 (6.5%)	Impact on impression of program	Very negatively/negatively	20 (71.4%)
			No impact	7 (25.0%)
			Very positively/positively	1 (3.6%)
		Effect on actual ranking	Much less/less likely to rank it highly	14 (50.0%)
			No effect	10 (35.7%)
			Much more/more likely to rank it highly	4 (14.3%)
Implication of matching into program if ranked highly	161 (37.2%)	Impact on impression of program	Very negatively/negatively	13 (8.1%)
			No impact	43 (26.7%)
			Very positively/positively	105 (65.2%)
		Effect on actual ranking	Much less/less likely to rank it highly	4 (2.5%)
			No effect	89 (55.3%)
			Much more/more likely to rank it highly	68 (42.2%)
Inquiry about other locations interviewing or interviewed at	261 (60.3%)	Impact on impression of program	Very negatively/negatively	139 (53.3%)
			No impact	121 (46.4%)
			Very positively/positively	1 (0.4%)
		Effect on actual ranking	Much less/less likely to rank it highly	59 (22.6%)
			No effect	200 (76.6%)
			Much more/more likely to rank it highly	2 (0.8%)
Asked to provide a commitment to a specific program before Match day	17 (3.9%)	Impact on impression of program	Very negatively/negatively	7 (41.2%)
			No impact	6 (35.3%)
			Very positively/positively	4 (23.5%)
		Effect on actual ranking	Much less/less likely to rank it highly	8 (47.1%)
			No effect	6 (35.3%)
			Much more/more likely to rank it highly	3 (17.6%)
Pressure to provide a statement of interest to programs outside of geographic area	202 (46.7%)	Impact on impression of program	Very negatively/negatively	45 (22.3%)
			No impact	151 (74.8%)
			Very positively/positively	6 (3.0%)
		Effect on actual ranking	Much less/less likely to rank it highly	22 (10.9%)
			No effect	170 (84.2%)
			Much more/more likely to rank it highly	10 (5.0%)
Breach of the NRMP 2018 Match Participation Agreement	135 (31.2%)	Impact on impression of program	Very negatively/negatively	85 (63.0%)
			No impact	49 (36.3%)
			Very positively/positively	1 (0.7%)
		Effect on actual ranking	Much less/less likely to rank it highly	51 (37.8%)
			No effect	83 (61.5%)
			Much more/more likely to rank it highly	1 (0.7%)
Other behavior perceived to have ignored the spirit of the Match	71 (16.4%)	Impact on impression of program	Very negatively/negatively	53 (74.6%)
			No impact	14 (19.7%)
			Very positively/positively	4 (5.6%)
		Effect on actual ranking	Much less/less likely to rank it highly	41 (57.7%)
			No effect	23 (32.4%)
			Much more/more likely to rank it highly	7 (9.9%)

Multivariate logistic regression was performed using the demographic variables as predictors with the response variables being each of the nine evaluated interview pressures: P.1: felt pressure to reveal ranking; P.2: explicitly asked to reveal ranking; P.3: implied matching if program was ranked highly; P.4: asked about other interview locations; P.5: asked for a commitment; P.6: pressured to send statement of intent; P.7: asked about partner’s interview; P.8: breached NRMP agreement; and P.9: other behavior ignoring spirit of the Match. Specialty competitiveness remained significantly predictive for P.1 (p = 0.03), P.2 (p = 0.01), P.4 (p = 0.001), and P.8 (p = 0.04). Number of interviews remained significantly predictive for P.3 (p = 0.01) and P.8 (p = 0.01), and medical school region remained significantly predictive for P.6 (p = 0.04). P.5, P.7, and P.9 had no significant predictive variables in the multivariate analysis (Table 2).

**Table 2:** Multivariate Regression Table 2 describes the statistically significant results of the multivariate regression, showing the factors that remained independently predictive of Match violations.

Match Violation	Specialty Competitiveness	Number of Interviews	Medical School Region
P.1: felt pressure to reveal ranking	p = 0.03		
P.2: explicitly asked to reveal ranking	p = 0.01		
P.3: implied matching if program was ranked highly		p = 0.01	
P.4: asked about other interview locations	p = 0.001		
P.5: asked for a commitment			
P.6: pressured to send statement of intent			p = 0.04
P.7: asked about partner’s interview			
P.8: breached NRMP agreement	p = 0.04	p = 0.01	
P.9: other behavior ignoring spirit of the Match			

Qualitative results 

Of the 433 survey respondents, 31.2% (n = 135) shared “comments regarding the Match interview process that negatively impacted [their] impression of a program” and 28.4% (n = 123) provided “comments regarding the Match interview process that positively impacted [their] impression of a program.” Twenty-seven percent (n = 117) gave suggestions to improve the interview process. All of the comments comprised three overarching themes: interview experience (e.g., scheduling, logistics, and interviewer preparation), professionalism (e.g., honesty and integrity; approachability of interviewers; respect for the spirit of the Match; and avoidance of personal, geographic, and ranking questions), and PIC.

Regarding interview experience, applicants preferred to interview at programs where the residents were enthusiastic and involved during the interview process. Applicants also preferred interviewers who are well prepared and familiar with their application, as demonstrated by the following quote:

“The thing that had the greatest negative impact on my impression of a program was how unprepared interviewers were for my interview. Some had obviously never seen my application, CV, or essay…”

Regarding professionalism, applicants disliked programs that made negative comments about other programs, asked personal questions (about marital status, family planning, or religion), or probed them to divulge the other places they applied, interviewed, or ranked. Several applicants reported that certain questions caused them to rank programs lower. One applicant said:

“… Any sort of questioning that was not friendly or just combative, that program was ranked much lower.”

Another reported:

“… Unethical questions about me being a woman with children going into surgery placed a program at the bottom of my rank list despite enjoying my time with the residents and program director.”

Yet another said:

“… what I did have a problem with was being asked point blank if my home program, where I rotated, or where I completed a research year, offered me an interview (which happened frequently).”

Regarding PIC, applicants overwhelmingly agreed that PIC should either be eliminated or that programs should set clear expectations for PIC.

Representative quotes include:

“… Some of my [PIC] from a program was toeing the line of breaking the Match rules on their part (although this program was ranked highly for me, so I was happy to hear the things I heard from them). Overall, I think virtually all [PIC] undermines the spirit of the Match.”

“Even without explicitly asking for how you ranked them, I disagree with the use of ‘ranked to match’ or ‘highly ranked’ communications from [program directors] … I think there should be no [PIC].”

“… faculty demanded letters of intent proving I was ranking them first in order to be ranked competitively … I mean, I was interested in their programs and would have happily gone there. But if they’re ranking applicants based on how likely they think that applicant is to come to their program…”

Many applicants reported appreciating programs that did not place pressure on applicants to engage in PIC:

“Programs that openly endorsed the Match process, saying they believe it works and puts people where they belong was a huge positive to me. I really appreciated those who went out of their way to say [PIC] was discouraged and did not influence their decisions. I felt those program directors were more trustworthy and honestly and truly wanted the best for their program.”

Applicants’ suggestions included:

“Every program should have a written post-interview contact policy clarifying if they do or do not want thank you letters, letters of interest, etc. and what the program’s policy is on sending [PIC]”

“No [PIC] … follow NRMP guidelines and make sure all interview committee members are knowledgeable of said guidelines.”

Table 3 summarizes applicants’ suggestions for improvement.

**Table 3:** Advice for Residency Program Directors Based on 2018 Residency Match Applicant Comments (n = 117)

Advice Theme	Recommendations	Summative Match Applicant Quotes
Interview Experience	Improve pre-interview communication and scheduling	“The more communication about what to expect throughout the process, the better!”
	Review applicants’ application and CV prior to one-on-one interview and ask personalized interview questions	“Lack of organization, interviewers [who] clearly hadn’t read my application, presence of program coordinator at pre-interview dinner, [and] lack of residents/few residents at dinners/lunches during [the] interview process [gave a negative impression of a program].”
	Ensure a sufficient number of residents are available and enthusiastic during interviews	“[I appreciated] programs that were prepared and organized beforehand with the questions each interviewer was going to ask me [as well as] questions regarding my personal accomplishments, interests, and goals.”
		“The only negative experience I had was at a program where it was evident that the interviewer (associate program director) had not looked at my file prior to my interview … it felt very impersonal and unprofessional … I loved the program on paper, but ended up ranking it very low due to my negative experience with the interviewer…”
Post-Interview Communication (PIC)	Discourage letters of intent	“Even without explicitly asking for how you ranked them, I disagree with the use of ‘ranked to match’ or ‘highly ranked’ communications from [program directors] … I think there should be no [PIC]. We as applicants also feel pressure to send [number one] emails, which [we] shouldn’t be.”
	If unable to eliminate PIC, then communicate clear expectations for PIC and make every effort to ensure it has no impact on ranking	“[I] really liked programs that said we do not contact anyone post interview and although we welcome any questions we do not need any thank you emails or letters. It was really nice not to have to play that game.”
		“Every program should have a written post-interview contact policy…”
		“I think the NRMP should ban [PIC] between programs [and] applicants. A few programs asked applicants not to contact them [and] I appreciated that … without a strict rule against [PIC], the Match encourages dishonesty and punishes many good applicants unwilling to engage in unethical behavior.”
Professionalism and Respect for the Match	Avoid inappropriate questions (about marital status, family planning, rank list, interview locations, etc.)	“Being a woman interviewing for orthopedics I received several sexist comments, some more innocent/well-intending than others. In particular some interviewers would imply or state that they were only planning to match one woman…”
	Rank applicants based on their own merit (i.e., grades, Step scores, interview, etc.) and not their level of interest in your specific program (i.e., likeliness to attend your program)	“Programs that spoke negatively about other programs left me with a bad impression in addition to programs that asked me where else I applied.”
	Educate all interviewers on the Match Participation Agreement	“… follow NRMP guidelines and make sure all interview committee members have knowledge of said guidelines.”
		“I had a program director spend the majority of our interview pushing me for more specific details on where I had applied and what programs I was debating ranking highly. They also specifically asked me what I thought of my home program … it very negatively impacted my ranking of the program.”

## Discussion

Our results show that Match violations still commonly occur and can negatively affect applicants’ impression and impact the ranking of programs. We expand upon earlier literature by assessing both overt match violations and those actions that ignore the spirit of the Match without explicitly violating the Match Participation Agreement (MPA) [5,6,8-10]. Our findings are similar to those of Hern et al. [6] who reported that during the 2007 NRMP Match, approximately one in five applicants were asked for commitments to rank programs highly during interview season, leaving many applicants feeling uncomfortable and less likely to rank the program highly. Follow-up work by the same author [9,10] was similarly consistent with our findings. They noted that asking students for a commitment caused discomfort and decreased program rank, as did asking about age, gender, marital status, parental status, plans for childbearing, ethnicity, religion, and sexual orientation.

Applicants in this cohort were frequently asked where else they interviewed (60.3%, n = 261), which is similar to another study of a large single academic institution showing that 72% of applicants were asked at least once about their other interviews [11]. Other program actions - including pressure to reveal ranking, asking to provide a commitment to a specific program before Match Day, and additional behavior perceived to ignore the spirit of the Match - left applicants with varying degrees of negative program impressions and resulted in 31.7%-57.7% of those applicants being less to much less likely to rank those programs highly. These actions are not isolated; many applicants experienced multiple Match violations. While students from all demographic categories are susceptible to match violations, certain students did report them with higher frequency. Higher Step 2 scores, greater number of interviews, and application to a surgical specialty were all associated with higher likelihood of experiencing a match violation. We hypothesize that interviewers in competitive specialties utilize these tactics in an attempt to narrow the qualified applicant pool, given the generally lower number of available positions in these fields. However, our findings suggest that such measures may be counterproductive for these programs by driving away applicants rather than attracting them.

Notably, a majority (65.2%) of applicants were positively to very positively impacted by programs implying they would match at said program if it was ranked highly, and 42.2% were more likely to much more likely to rank the program highly. It may be tempting for programs to utilize this tactic to increase their likelihood of matching desired applicants. However, we feel that this is ethically inappropriate, and programs should remain cognizant of the fact that this is still a Match violation and can trigger disciplinary action from the NRMP. 

The program director was the most common perpetrator of Match violations, closely followed by other faculty, residents, and program coordinators. As the leader of the resident selection process, it is likely that the attitudes of the program director permeate throughout the program. Our findings suggest that positive changes would be most impactful if encouraged and spread by the program director.

Our collection and examination of qualitative data expand upon the aforementioned insights gained from our quantitative data. This study is unique in its emphasis on qualitative data from students entering a broad range of specialties. To our knowledge, there are only two other studies to date that examined student comments related to unethical match behavior, both of which were specialty-specific [5,12]. In their comments, students overwhelmingly expressed frustration with their interview experiences, program professionalism, and PIC.

Students described negative perceptions of programs that appeared disorganized and ill-prepared, asked violating questions about personal life or questions that appeared generally disrespectful, and were not familiar with their applications. The underlying implication is that students felt they put a great deal of effort into attending and preparing for each interview and took care to treat others respectfully, but these courtesies were not reciprocated. These findings suggest that better preparation prior to interviews on behalf of the interviewer, as well as maintenance of a professional and respectful interview environment, would improve applicants’ perceptions and ranking of a given program. These are easily made improvements if one is cognizant of them, and they can likely improve a program’s reputation and advance their goal of recruiting desirable applicants.

PIC was also identified as an area of concern. Students felt that it ignores the spirit of the Match, creates confusion, and adds stress to the application process. Students also negatively perceived being asked to commit to a program after the interview. Even positive PIC from programs, such as telling an applicant that they are highly ranked, was frequently viewed as confusing. The underlying implication was that students felt these messages could be disingenuous and unfairly pressured them to respond in kind. Indeed, several specialty-specific studies found that PIC impacted applicant and program rankings for a noteworthy minority of individuals (approximately 20%-30%) [13-16], which likely underlies the stress of PIC on both parties. Our respondents overwhelmingly echoed the sentiment in the available literature that PIC, in its current form, should be eliminated completely [11,17-19]. If PIC is to continue, applicants prefer that programs create and adhere to strict policies to eliminate ambiguity. In accordance with the preferences of these students, we recommend programs simply cease to engage in this practice by refraining from sending PIC and clearly telling students that none is expected of them as well.

Limitations of our study include the potential for recall bias as well as negativity bias; applicants with more negative match experiences may have been more likely to respond to the survey than those with positive experiences. Another limitation is a relatively small sample size. We were unable to send our survey directly to applicants’ personal emails and had to rely on third parties. Thus, we are unsure of exactly how many applicants received the survey, as some intermediaries may have chosen not to forward it. For this reason, we were unable to calculate an exact response rate. Despite this limitation, we believe our results are valid as they both are consistent with and expand upon prior studies [6,9,10]. The responses we received were predominated by applicants in the Southern region (54.7%, n = 237), though these applicants were otherwise nationally representative in terms of gender, medical school type, and the United States Medical Licensing Examination (USMLE) scores. Clear trends emerged, and the free-response questions provided valuable and unique insight into how applicants perceive programs and the interview process. The primary strength of this study is our collection of a large breadth of both quantitative and qualitative data from applicants in all specialties and regions, making this broad study unique in the available literature.

## Conclusions

Match violations continue to occur. The most frequently reported Match violations include asking about other interviews, explicitly pressuring applicants to reveal the locations of other interviews, and asking applicants to provide a commitment prior to Match Day. Additionally, applicants described several program behaviors which, while not explicitly forbidden, were interpreted to violate the spirit of the Match. These included unethical behavior, pressure to attend a second-look visit, inappropriate PIC, or other undue pressure.

With the notable exception of communication implying that applicants would match into a program, applicants overwhelmingly view programs that commit Match violations negatively and often rank these programs lower as a result. PIC in particular was consistently identified as unwelcome and confusing. Many applicants favored either establishing explicit PIC expectations or eliminating it entirely. The majority of Match violations were committed by Program Directors, and these leaders are uniquely positioned to lead the reform of the residency interview process. Based on these results, elimination of Match violations would likely be beneficial to both applicants and programs.
